# Combining Indoor Positioning Using Wi-Fi Round Trip Time with Dust Measurement in the Field of Occupational Health

**DOI:** 10.3390/s21217261

**Published:** 2021-10-31

**Authors:** Hajime Ando, Shingo Sekoguchi, Kazunori Ikegami, Hidetaka Yoshitake, Hiroka Baba, Toshihiko Myojo, Akira Ogami

**Affiliations:** 1 Department of Work Systems and Health, Institute of Industrial Ecological Sciences, University of Occupational and Environmental Health, Japan, 1-1, Iseigaoka, Yahatanishi-ku, Kitakyushu, Fukuoka 807-8555, Japan; s-sekoguchi@med.uoeh-u.ac.jp (S.S.); kikegami@med.uoeh-u.ac.jp (K.I.); hide-yoshitake@med.uoeh-u.ac.jp (H.Y.); h-baba@med.uoeh-u.ac.jp (H.B.); gamisan@med.uoeh-u.ac.jp (A.O.); 2 Department of Environmental Health Engineering, Institute of Industrial Ecological Sciences, University of Occupational and Environmental Health, Japan, 1-1, Iseigaoka, Yahatanishi-ku, Kitakyushu, Fukuoka 807-8555, Japan; tmyojo@med.uoeh-u.ac.jp

**Keywords:** indoor positioning, Wi-Fi RTT, occupational health, wearable particle monitor

## Abstract

Monitoring of personal exposure to hazardous substances has garnered increasing attention over the past few years. However, no straightforward and exact indoor positioning technique has been available until the recent discovery of Wi-Fi round trip time (Wi-Fi RTT). In this study, we investigated the possibility of using a combination of Wi-Fi RTT for indoor positioning and a wearable particle monitor (WPM) to observe dust concentration during walking in a simulated factory. Ultrasonic humidifiers were used to spray sodium chloride solution inside the factory. The measurements were recorded three times on different routes (Experiments A, B, and C). The error percentages, i.e., measurements that were outside the expected measurement area, were 7% (49 s/700 s) in Experiment A, 2.3% (15 s/660 s) in Experiment B, and 7.8% (50 s/645 s) in Experiment C. The dust measurements were also recorded without any obstruction. A heat map was created based on the results from both measured values. Wi-Fi RTT proved useful for computing the indoor position with high accuracy, suggesting the applicability of the proposed methodology for occupational health monitoring.

## 1. Introduction

The amount of dust that workers are exposed to inside factories in Japan is assessed through workplace environment measurements. However, this method has some shortcomings; for instance, it tends to overlook the exposure status of workers when they work while moving. Therefore, an amendment to the Work Environment Measurement (WEM) Standards has been suggested to enable personal exposure measurements instead of conventional workplace environment measurements for certain hazardous substances [1]. Therefore, personal exposure monitoring is expected to be used at workplaces where it is difficult to measure hazardous substances, such locations where (a) temporary or short-term work is conducted, (b) the source of dust is moving, or (c) work is done outdoors. Due to recent advances in sensor technology, compact PM2.5 sensors have been developed. These were originally intended for measuring air quality, but they can also be used for evaluating personal exposure to dust. Therefore, we developed a wearable particle monitor (WPM) that uses a commercial sensor (HPMA115C0-003, Honeywell International, Inc., Morristown, NJ, USA). Even though the sensor has not been calibrated under strict protocols, its module (manufactured by Honeywell) has been reported to be accurate [2]. Given that this product has not been officially calibrated, it cannot be formally used for workplace environment measurements, but it can suffice for calculations in a simulated environment.

When conducting personal exposure measurements, it is necessary to record actions and observations [3]. However, recording actions and observations accurately while conducting measurements is difficult because it requires a dedicated observer. In an industrial environment, the nature of the work being performed, such as welding, cutting, or painting, is considered to be related to the location of the work. Thus, if the time and location are known, it is possible to estimate the nature of the work. Obviating the need for an observer will considerably reduce labor costs. The Global Navigation Satellite System (GNSS), represented by the Global Positioning System (GPS), is the standard technology for outdoor positioning [4]. However, such technologies cannot be utilized for indoor positioning because satellite signals cannot be received indoors. Instead, several nonstandard methods, such as Bluetooth low-energy beacons [5,6,7], Wi-Fi signals [6,8], and various sensor-based devices [6], are used. However, these methods are limited in terms of accuracy; hence, several measures have been proposed to address the deficiencies of such techniques. In 2016, Wi-Fi round trip time (RTT) distance measurement was standardized in IEEE 802.11mc as a high-accuracy method [9]. Using Wi-Fi RTT, indoor position can be computed accurately by calculating the distance from the arrival time of the radio waves, rather than by estimating the distance from the radio wave strength, as in conventional methods. The Wi-Fi RTT protocol enables positioning without synchronizing the internal clocks of two devices (Smartphone and WAP, or two smartphones, etc.). This eliminates the need for high-precision clocks, such as cesium atomic clocks. The requisite hardware can also be easily installed using consumer Wi-Fi access points (WAPs). However, only a few reports on positioning using this standard have been published thus far, and these have been largely restricted to the field of engineering. Therefore, in this study, we investigated the possibility of utilizing Wi-Fi RTT and WPM for monitoring of occupational health at industrial sites.

## 2. Materials and Methods

**Figure 1:**
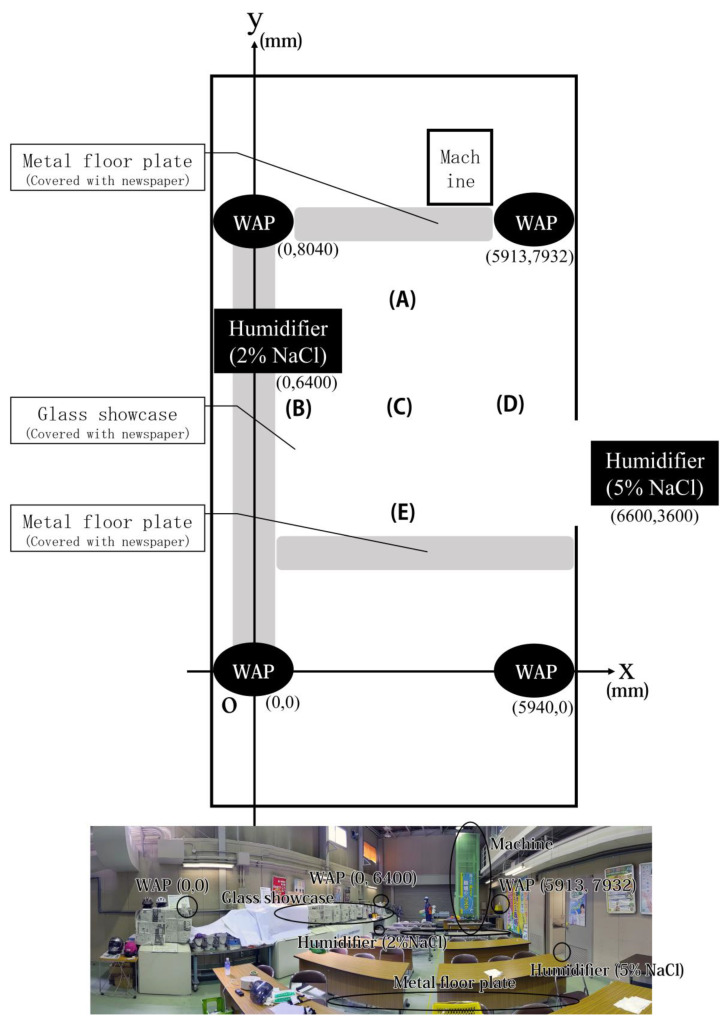
Overview and photographs of the model factory. The schematic diagram indicates the positions of the measurement points for accuracy verification, the coordinates measured by the laser rangefinder are: (A) (2900, 6500); (B) (1900, 3000); (C) (3000, 3200); (D) (5700, 2800); and (E) (3100, 800) (WAP denotes the Wi-Fi access point). The photo below was taken from, approximately the coordinates (3000,0). The humidifier is not installed; the WAP installed at the coordinates (5940, 0) is out of the field of view of the camera; the metal floor near point (A) is hidden from view in the photo; the metal floor near point (E) is roughly in the position indicated. The glass showcase is covered with paper or cloth, and the metal floor is not covered.

This experiment was conducted in August 2020 in a multipurpose simulated factory at the University of Occupational and Environmental Health, Japan. The overall layout is illustrated in Figure 1. The simulated factory was constructed to resemble the structure of a typical Japanese factory. Desks and chairs were set up for lectures, and glass showcases were setup for display. Such glass showcases are often used in actual factories as exhibits for visitors. In some places, desks and chairs were lined up, as shown in Figure 1, representing instances where work was done by hand rather than machines.

### 2.1. Indoor Positioning

**Figure 2:**
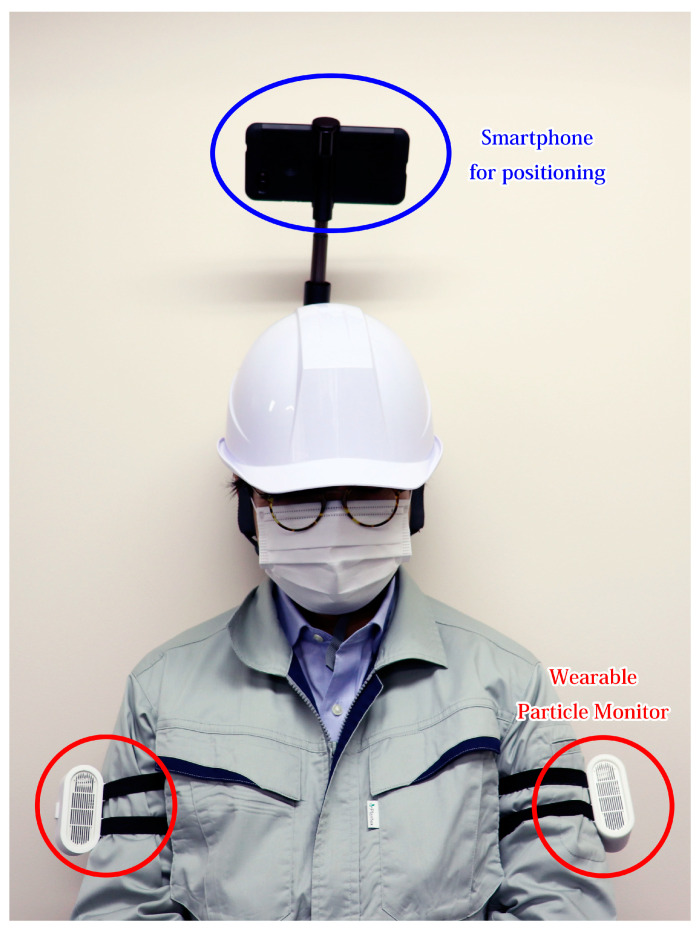
Photograph of the measurer. A wearable particle monitor was worn on both upper arms. A smartphone for indoor positioning was attached to the helmet using a selfie stick.

Wi-Fi RTT was used to compute the indoor position. We used a Pixel 4 smartphone (Google LLC, Mountain View, CA, USA) [10], a Nest Wi-Fi (Google LLC, Mountain View, CA, USA) router [11], and three expansion points as WAPs. We were unable to find any existing application for indoor positioning that matched the requirements of this study. Therefore, we modified an open-source (MIT License) [12] software program developed by Darryn Campbell [13]. The major modifications included changing the coordinate system of the WAP and the addition of a function to save the log. Moreover, we used the developer mode to bypass the restriction on the number of Wi-Fi scans performed by the Android OS. This application measured the distance by Wi-Fi RTT to each WAP every 500 ms. The distance between the smartphone and WAPs was obtained from the Android OS API, and no correction was applied. Utilizing the distance obtained, the position was calculated by trilateration, and the time and coordinates were recorded. The coordinates of the WAP locations were determined based on measurements from a GLM 50 C Professional Laser Rangefinder (Robert Bosch GmbH, Gerlingen, Germany) [14]. The heights of the WAP locations were approximately 1500 mm from the floor. Given that the height remained the same for all the WAPs, the change in Z coordinate was negligible, and therefore was not considered in this study. The WAPs were placed as shown in Figure 1. The preliminary experiments showed the positioning to be inaccurate in the area around a glass showcase and the area where the floor material was made of steel. Hence, these areas were covered with paper or fabric. In a previous study, it was reported that the accuracy of the positioning varied with the direction of movement because the body acts as a shield [15]. Therefore, a smartphone mounted on a selfie stick was fixed to the helmet for indoor positioning. A photograph of the measurer with the gadget and WPM is shown in Figure 2. Moreover, a wearable particle monitor was affixed on both of the upper arms. 

### 2.2. Confirmation of Measurement Accuracy

Prior to this experiment, we examined the measurement accuracy of our gadget for the Wi-Fi RTT measurements at all location points from points (A) to (E) in Figure 1. Measurements were acquired from each of the points for a duration of one minute. The coordinates measured by the laser rangefinder were used as the standard, and the deviation of the measurements computed using Wi-Fi RTT was calculated.

### 2.3. Dust Measurement

The WPM was used for the dust exposure measurements. The WPM utilized an HPMA115C0-003 sensor, which was connected to the smartphone via Bluetooth. The dust concentration per second obtained by the sensor was received by the smartphone, and recorded along with the time. These readings are recorded in the WPM internal memory. However, missing values occur in rare cases due to telecommunication errors. Therefore, we analyzed the data using the internal memory records. This sensor is capable of outputting values for PM1, PM2.5, PM4, and PM10, but the other three values were estimated from the PM2.5 value. The measurable range of PM2.5 is from 0 to 1000 μm/m^3^, which is sufficient for general work environments. The detailed specifications of the WPM and sensor modules are shown in Table 1 and Table 2.

One unit of the WPM was affixed to the left, and another unit was affixed to the right upper arm of the person performing the measurement. Given that there was no source of dust in the simulated factory, two ultrasonic humidifiers were installed, and a sodium chloride solution (2% and 5%) was sprayed. Commercial salt was used as the solute, and pure water was used as the solvent. The measurements were acquired every second, and cases with zero readings or no records were excluded. After ventilating the room for each measurement, the humidifier was turned on, and measurement readings were taken after a duration of (at least) 3 min.

### 2.4. Measurement Procedure

**Figure 3:**
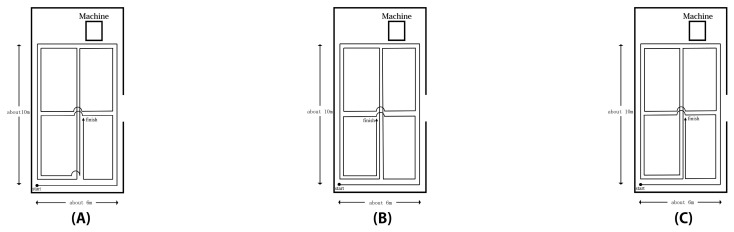
Walking path during the measurement. Considering the possibility of the direction of movement affecting the measurement results, the experiment was conducted for three different walking routes: (**A**) Experiments A; (**B**) Experiment B; (**C**) Experiment C. The start and end points of the three experiments were the same, but the turning directions along the routes were different, and the directions of travel were different.

Measurements were performed three times for each different route. The measurer walked the designated route at a steady pace of approximately 7.62 m/min. Figure 3 shows the walking route during the measurement.

### 2.5. Creation of Heat Maps

A heat map was created combining the positioning information computed using Wi-Fi RTT and the results of dust measurements by the WPM. The location information was calculated as the median value of the X- and *Y*-axis coordinates every second. The heat map was created by excluding the coordinate values outside the original circumference range (a rectangular range consisted of four points: (0, −1000), (6000, −1000), (0, 9000) and (6000, 9000)). The dust concentration was determined by using the median of the measured values in the area. The heat map was created using Origin 2020 (64-bit, SR1 version 9.7.0.188, OriginLab Corporation, Northampton, MA, USA) [16].

## 3. Results

**Figure 4:**
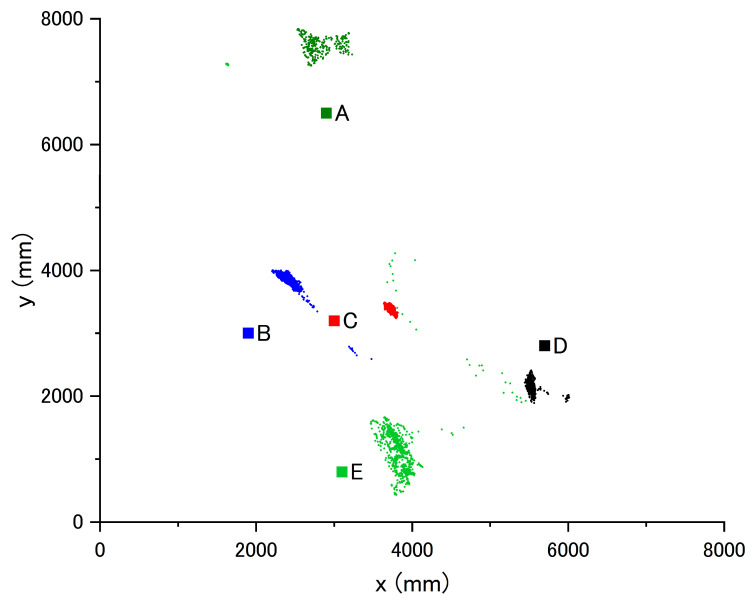
Results of accuracy verification. A–E shows each point of (A–E) shown in Figure 1. The small dots indicate the position measured by the Wi-Fi RTT at each measurement point. The squares indicate the actual position of each measurement point. The measurement results from the Wi-Fi RTT are concentrated in a specific range at each point, and the precision is considered to be relatively high. The level of accuracy is considered to be sufficient for the purpose of this study.

The results from the accuracy verification procedure are shown in Figure 4 and Table 3. The mean (standard deviation) from the laser rangefinder position at each point was 1087.9 (133.7) mm at point A, 1000.6 (63.7) mm at point B, 748.3 (31.5) mm at point C, 669.2 (94.3) mm at point D, and 918.4 (396.8) mm at point E. The net error in this measurement system was approximately 1 m.

**Figure 5:**
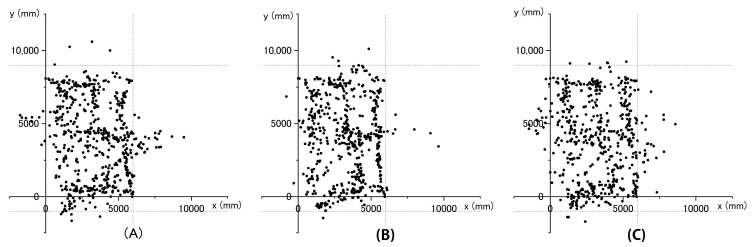
Indoor positioning results. Each dot indicates the position coordinates computed by the Wi-Fi RTT. The location information was calculated as the median value of the X- and *Y*-axis coordinates every second. The range enclosed by the dotted line and the *Y*-axis is the walking range. (**A**–**C**), indicate Experiments A, B, and C, respectively.

The results of the indoor positioning are shown in Figure 5. Some measurements recorded locations that could not possibly occur, such as behind the glass showcase or behind the wall, which were outside of the rectangle comprised of the coordinate locations “(0, −1000), (6000, −1000), (0, 9000), (6000, 9000).” In these cases, the error percentages were recorded as 7% (49 s/700 s) in Experiment A, 2.3% (15 s/660 s) in Experiment B, and 7.8% (50 s/645 s) in Experiment C. We obtained the same path position indoors for the measurer using the Wi-Fi RTT. Although there was a certain amount of error in verifying the accuracy, we were able to measure the movement path continuously without interruptions. The experiments were conducted on three different walking routes, and no remarkable differences were observed in the positioning results obtained from the Wi-Fi RTT.

**Figure 6:**
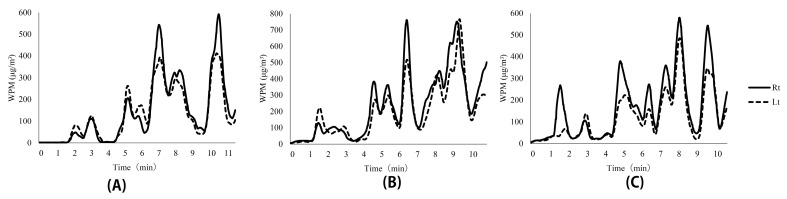
Results of dust measurements (Rt, right-hand side and Lt, left-hand side). (**A**–**C**) indicate Experiments A, B, and C, respectively. The source of the dust was located on the right side of the walking path. High values were reasonably predominant in the right-arm WPM.

The results of the WPM measurements are shown in Table 4 and Figure 6.

**Figure 7:**
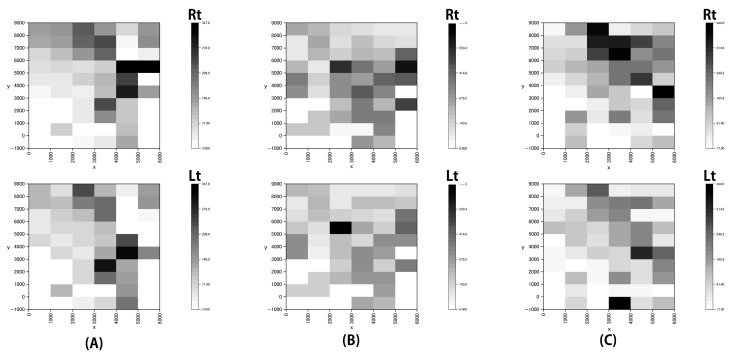
Heat maps. (**A**–**C**) indicate Experiments A, B, and C, respectively. The color of each area indicates the median dust concentration in that area (Rt, right-hand side and Lt, left-hand side). It was confirmed that the dust concentration near the humidifier with a 5% saline solution at (6600, 3600) was higher than those from the other areas. This was considered to reflect the dust emission in the experimental environment. In addition, it was confirmed that the dust concentration near the coordinate location ranges from (x = 2000–4000, y = 7000–9000) was high. In this experiment, the humidifier was installed at (0, 6400) and (6600, 3600) and acted as a dust source. This resulted in a gap in the heat map between the dust source and the areas with a high concentration of dust.

Figure 7 shows the heat map created by combining the two datasets.

## 4. Discussion

The error in this measurement system was approximately 1 m. In a previous study, it was reported that the average measurement error of the Wi-Fi RTT was 1.4 m in an outdoor experiment where the measurement points were set in a grid pattern at equal intervals [17]. Another study reported the average measurement error of the Wi-Fi RTT to be 0.10 m in an outdoor experiment, and 0.23 m in an indoor experiment, where the measurement points were set along a straight line at equal intervals [18]. Although the accuracy obtained in this study was less than that in the aforementioned studies, it is considered to be adequate for use in workplaces. The accuracy of the GNSS positioning using a smartphone has been reported to be 8–10 m [19], and the Wi-Fi RTT is expected to be more accurate than the GNSS, if configured properly. In this experiment, the maximum error was 3.5 m, which is considered to be suitable for use in occupational health. Further, the WAPs were installed at a higher density than those used for normal Internet connections. For positioning, three Wi-Fi signals should be received simultaneously. Thus, it may be necessary to install additional WAPs temporarily for positioning.

In the Wi-Fi RTT positioning, we obtained a trajectory similar to the walking route in all three cases. It was assumed that the human body influenced the Wi-Fi RTT positioning, as previously reported in [15]. To overcome this issue, the smartphone was held high above the head by means of a selfie stick fixed to the helmet. In addition, in this study, we covered the glass and metal parts in advance because they were found to reduce the accuracy of measurements in the preliminary investigation. In this regard, it was assumed that the reflection and refraction of radio waves caused by the glass and metal parts reduced the accuracy. Hence, it would be necessary to conduct tests in advance and resolve this issue before using the Wi-Fi RTT for measurements in actual workplaces. There have been attempts to create fingerprints using Wi-Fi RTT and machine learning [20]. Accordingly, the accuracy of this method is expected to improve as the technology develops. However, most of the validation studies on the accuracy of the Wi-Fi RTT have been conducted in empty plazas or ordinary offices, and therefore it is desirable to conduct these verification studies in industrial settings, such as factories. Although we did not verify the accuracy of the measurement while walking, it was presumed that there was a certain degree of accuracy given that the walking path was measured without any obstruction (Figure 5). Considering that the mesh of the heat map is 1 m, the measurement was conducted during walking at an extremely steady pace. In an actual work site, it is assumed that people move faster while walking around their workplace. Given that most of the work is done under stationary conditions and that the accuracy in such cases has been verified, this discrepancy is not expected to be a major problem. To expand the usage of indoor positioning, an additional study on accuracy during a worker’s movement in the workplace is required.

One of the advantages of the Wi-Fi RTT is that it can be used in local networks, whereas the GNSS requires orbital information from satellites to identify a particular location. Without the Internet, more than 30 s are required to obtain orbital information from GNSS satellites, and the accuracy is initially low [21]. Given that the Wi-Fi RTT requires only a WAP and a smartphone for positioning, it does not require this external connection or orbital information. Instead, the Wi-Fi RTT relies on the coordinate information of the WAP, which is not a concern because the coordinate information of the WAP is already known by the company. This can offer a great advantage in the field of occupational health given that external connections are often difficult to access inside factories. In this study, we used the Wi-Fi RTT in combination with dust measurement. Notably, the GNSS has already been used in various applications, such as geofencing and disaster prevention [22]. There are various other applications possible if highly accurate positioning by GNSS is achieved indoors.

Unlike existing methods, Wi-Fi RTT calculates the distance based on the arrival time of the radio wave rather than its strength. Theoretically, the radio wave strength attenuates in proportion to the square of the distance, but in reality, this does not hold true owing to the effects of various noise sources. In contrast, the Wi-Fi RTT uses the arrival time, and thus is considered to be less susceptible to noise. However, both methods are affected by the reflection, refraction, and diffraction of radio waves; hence, this should be considered. In this study, the smartphone was placed on a helmet to reduce these effects. However, considering safety protocols, it may not be realistic to use this method at a workplace. A more stable fixture should be considered along with technological advances to make the gadget portable. In this study, we set the height of all the WAPs and smartphone to be almost the same to disregard the variation in *Z*-axis. However, if each WAP is set higher, such that the radio wave can reach the smartphone directly without being blocked, more stable positioning may be possible, but this hypothesis has to verified. 

Although there is no specific limit to the number of times that the Wi-Fi RTT can be used, the Android OS specification sets a limit to the number of times that the Wi-Fi APs can be scanned [23]. In addition, there is a limit to the number of times that the Wi-Fi RTT can be used in the background and for other location information; however, no such limit exists for its use in the foreground [24]. It was reported that the Wi-Fi RTT consumes excessive battery and is associated with certain privacy issues. When the restriction is enabled, scanning for Wi-Fi APs is suppressed. This particular aspect of the operating system could have hindered us from obtaining an accurate indoor position. We overcame this hurdle by using the developer mode, which allows the required features. Restrictions may change over time and should be monitored carefully. In actual occupational health settings, the use of dedicated devices will be considered to ensure privacy. If indoor positioning becomes popular, dedicated terminals may be developed. The effective range of Wi-Fi signals is not wide; hence, the reach of the signal is limited to the inside of the premises in a large space such as a factory. Internet connection is not necessarily required for positioning. In this experiment, neither the access point nor the smartphone was connected to the Internet. In terms of usability, it is efficient to use already installed access points for positioning. However, considering privacy, it is possible to isolate the access points from the external network. Companies should determine the method that is appropriate for application.

The Wi-Fi RTT may be cost effective if the existing equipment meets the requirements. In terms of positioning accuracy, ultra-wideband (UWB) is considered to be superior to Wi-Fi RTT; a study on positioning inside a small room (approximately 8 × 3 m) reported an accuracy of 9.8–40.3 cm [25]. However, UWB is yet to gain widespread acceptance, although it is already being used in some smartphones, such as the Apple iPhone 11 Pro. Wi-Fi facilities are already widespread; if RTT is supported, it may be possible to use existing facilities for inexpensive and quick deployment. This is extremely important, especially in large workplaces. However, in Japan, UWB is limited in terms of the number of bands that can be used outdoors. The ban on the usage of some frequency bands outdoors was lifted in 2019; however, some bands are still only allowed indoors. Caution must be exercised while using these bands outdoors. There are fewer restrictions on Wi-Fi because it is more widely used than UWB.

The WPM was developed as a highly portable dust monitor. Although it is limited in functionality and operating time as compared with that used in a previous study [26], it is designed to be more compact. For workplace measurements, it is important for the system not to interfere with work, in addition to being small and lightweight. It also has a built-in LED that changes color depending on the dust concentration, and it can be operated as a stand-alone device when real-time measurements are not required.

In this study, the dust exposure on the WPM on the upper right arm was observed to be generally higher than that of the upper left arm. The mean of the WPM results for the right arm exceeded the mean of the WPM results for the left arm in all experiments. The humidifier was closer to the right side while walking, and the results were consistent with this observation. Looking over the heat maps of the three experiments, it can be seen that the dust concentration near the humidifier with a 5% saline solution at (6600, 3600) was higher than those in the other areas. This was considered to be a reflection of the dust emission in the experimental environment. In addition, it was confirmed that the dust concentration near the coordinate ranges, (x = 2000–4000, y = 7000–9000) was high. In this experiment, the humidifiers, which can be considered as the dust sources, were installed at (0, 6400) and (6600, 3600), and this has resulted in a gap between the dust source and the areas with high concentration of dust in the heat map. This discrepancy can be explained by errors in position measurement. The ventilation fan may have interfered with the dust exposure information in areas beyond the measurer’s physically reach. It is possible to interpret the results by assuming that the influence of the error in positional measurement was not significant, i.e., the heat map accurately reflected the dust concentration in the environment to some extent. This variation could be explained by considering the following: First, the dust might have been carried away by the movement of the measurer to the location in question. This aspect was visually confirmed during the experiment (including the preliminary experiment), although it has not been precisely verified or evaluated. Second, there is a possibility of the dust being shifted to the location in question by the operation of the ventilation fan in the simulated factory. By creating a heat map of the dust levels, we were able to visually determine the dust emissions in the environment. It is likely that the high-dust concentrations formed at a distance from the dust-emission source were not fully identifiable by the usual measurement in the workplace environment. In this study, we used a 1 m mesh, which was more detailed than the 6 m mesh used for the measurements in the workplace environment. Although it was not possible to measure all the points simultaneously, we were able to obtain data at many points.

The ability to visually confirm the distribution of dust by combining location information and dust meter results is considered to be extremely useful in monitoring industrial health activities. After the accident at the Fukushima nuclear power plant, some studies have sought to clarify the distribution of air dose rates by mapping concentration/amount of hazardous substances in combination with location information [27,28]. A previous study investigated the measurement of dust concentration by fabricating an instrument equipped with GNSS and a low-cost dust sensor [26]. However, these reports used the GNSS in outdoor environments. In the past, indoor positioning was limited by the available technology. Therefore, to create these heat maps, it was necessary to determine the position from video camera images, which was very complicated. In addition, personal exposure measurement sometimes involves synchronization of continuous recordings using a real-time monitor with a data logger and video images acquired during work to visually capture the changes in exposure that accompany work changes [29]. Work scenes were often filmed and analyzed, but recent studies have been conducted, wherein a small camera and small dust meter were attached to a helmet, and the camera and dust meter were synchronized to assess the dust emission status [30]. Although this approach has been in use for some time, it has not been extensively utilized in actual occupational health settings, often due to the fact that as the measurement time increases, it takes longer to check the video images during the analysis. This is especially true of continuous recordings of the exposure status and the synchronization of the video images. Therefore, it would be more practical to focus on situations associated with high exposures rather than checking the whole video stream during the entire measurement period. Our method has a significant advantage owing to the fact that we could get a snapshot of the entire workplace in a relatively short time. However, with the use of the method in our study, if a worker stays in the same place for a long time, the heat map will show only a single point; hence, it is necessary to limit the analysis duration or adjust the size of the heat map. If it is not possible to uniquely link the work location with the work content, a combination of video images along with our method can be considered. By combining indoor positioning technology, it will be possible to combine and analyze location and exposure information, which has previously been realized outdoors only.

This study had a few limitations. First, the measurement in this study was conducted using only one piece of equipment. Hence, we cannot generalize it for measurements obtained from other equipment. However, at present, only a few devices officially support Wi-Fi RTT in Japan. Moreover, the most commonly available devices are produced by Google. In particular, there are only four WAPs listed on the Android developer site as Wi-Fi RTT-compliant APs, and three of them are made by Google [31]. The fourth one is a foreign product and cannot be used because it does not have a technical standards compliance certificate in Japan. However, the number of compatible smartphones is gradually increasing. Second, we did not conduct simultaneous measurements of the measurer’s position using the standard method. Although no large discrepancies between the movement trajectories were assumed, it was not possible to quantitatively evaluate the degree of discrepancy. Additional evaluations are necessary, given that the required accuracy is likely to vary from workplace to workplace. Third, the simulated factory, used in this study, had fewer obstructions than a typical factory, and hence the measurement accuracy may have been overestimated. It is necessary to conduct surveys in more varied environments, and in certain cases, to combine methods such as fingerprinting for more robust results.

High-precision indoor positioning technology, along with the use of various sensors, is expected to have a wide range of applications, such as monitoring and evaluation of the workplace environment, and the observation of workers. Furthermore, this technology is of value in the field of occupational health because it can be used to conduct evaluations in various environments using different measuring instruments.

## 5. Conclusions

In this study, we conducted Wi-Fi RTT positioning and WPM dust measurement in a room to simulate a workplace. By combining both measurements to create a heat map, we were able to visualize the dust exposure and location during work, which was considered to be useful in monitoring occupational health. This method can also be applied to hazardous substances other than dust. The measurements obtained were considered to be sufficiently accurate for use in the field of occupational health, and further improvements in accuracy are expected with future technological advances.

Our results demonstrate the potential of indoor positioning technology and low-cost dust sensors to visualize the exposure status of hazardous substances. Confirmation of exposure status is crucial to reducing exposure to hazardous substances and preventing health problems, and we believe that our results present significant advancements to this end.

## Figures and Tables

**Table 1:** Specifications of wearable particle monitor.

Sensor	HPMA115C0-003 (Honeywell International, Inc., Morristown, NJ, USA)
Memory	Built-in 2 GB non-volatile memory
Time	Controlled by RTC
Record format	CSV
Alert	Color LED (changes color depending on dust concentration)
Transmission	USB or Bluetooth
Battery	LiPo battery 4+ h operation
Size	76.5 × 29.5 × 55.5 mm

**Table 2:** Specifications of sensor module.

Operation Principal	Laser Scattering
Detection	PM1.0, PM2.5, PM4.0, PM10
Unit	PM1.0, PM2.5, PM4.0, PM10 in μg/m^3^
Concentration range	From 0 to 1000 μg/m^3^
Response time	<6 s
Supply voltage	5 ± 0.2 V
Switching frequency max	100 kHz
Ripple amplitude max	20 mV
RMS noise max	1 mV (noise bandwidth 10 MHz)
Standby current	<20 mA (at 25 ± 5 °C)
Supply current	<80 mA (at 25 ± 5 °C)
Inrush current max	600 mA
Temperature: operating/storage	Operating, from −20 to 70 °C Storage, from −40 to 85 °C
Humidity: operating/storage	From 0% to 95% RH non-condensing
Operating time	Continuous mode: 10 years Intermittent mode, depends on duty cycle
Laser class	Laser class 1, IEC/EN 60825-1, 650 nm
ESD	±4 kV contact, ±8 kV air per IEC 61000-4-2
Radiated immunity	1 V/m (80 MHz to 1000 MHz) per IEC 61000-4-3 fast transient
Size	44 mm × 36 mm × 12 mm

**Table 3:** Measurement error at each point.

Point	N	Mean (mm)	SD (mm)	Min (mm)	Max (mm)
A	302	1087.9	133.7	779.8	1384.6
B	711	1000.6	63.7	889.8	1631.6
C	608	748.3	31.5	676.0	826.0
D	583	669.2	94.3	429.8	932.1
E	716	918.4	396.8	589.1	3539.5

SD, standard deviation.

**Table 4:** Descriptive statistics of dust measurement results.

WPM	N	Mean (μg/m^3^)	SD (μg/m^3^)	Min (μg/m^3^)	Max (μg/m^3^)
Experiment A right arm	700	142.7	151.5	1	593
Experiment A left arm	697	129.0	120.3	2	412
Experiment B right arm	660	234.8	197.8	5	761
Experiment B left arm	657	198.3	166.5	5	766
Experiment C right arm	645	163.1	142.3	7	579
Experiment C left arm	641	119.1	108.5	5	579

WPM, wearable particle monitor; SD, standard deviation.

## Data Availability

The data that support the findings of this study are available from the corresponding author upon reasonable request.
